# Higher target attainment for B-lactam antibiotics in patients with Gram-negative bloodstream infections when four times actual minimum inhibitory concentrations and epidemiological cutoff values are applied compared to clinical breakpoints

**DOI:** 10.1007/s10096-025-05068-x

**Published:** 2025-02-24

**Authors:** Ilja Areskog Lejbman, Gustav Torisson, Fredrik Resman, Fredrik Sjövall

**Affiliations:** 1 Department of Intensive and Perioperative Care, Skåne University Hospital, Carl-Bertil Laurells gata 9, Malmö, 205 02 Sweden; 2 Department of Infectious Diseases, Skåne University Hospital, Malmö, Sweden; 3 Department of Clinical Sciences, Lund university, Lund, Sweden; 4 Clinical Infection Medicine, Dept of Translational Medicine, Lund University, Lund, Sweden

**Keywords:** Beta-lactam antibiotics, Gram-negative bacteremia, Sepsis, MIC, ECOFFs, EUCAST clinical breakpoints, PK/PD

## Abstract

**Introduction:**

Beta-lactam antibiotics are essential in the treatment of Gram-negative bloodstream infections. The effect of beta-lactam antibiotics depends on the time of unbound antibiotic concentration above the minimal inhibitory concentration (MIC). An antibiotic concentration above MIC during the whole dosing interval (100% ƒT > MIC) has been suggested as a target for severe infections. The aim of the present study was to compare target attainment using targets derived from known MICs with standard generic targets.

**Methods:**

In this prospective, single-center study, adult patients with Gram-negative bloodstream infection treated with cefotaxime, piperacillin/tazobactam or meropenem were eligible for inclusion. Trough antibiotic concentrations were collected during a single dosing interval and actual MIC values for the antimicrobial agent against the infecting isolate were obtained using E-tests. Epidemiological cut off values, ECOFFs, were applied from European Committee on Antimicrobial Susceptibility Testing, EUCAST, tables for isolates within the wild-type distribution. Antibiotic concentrations were measured using Liquid Chromatography tandem Mass Spectrometry. Free concentrations were estimated based on total concentrations. Two targets based on actual MICs were assessed: free trough concentrations above (1) four times the actual MIC (100% ƒT > 4MIC) or above (2) the ECOFF (100% ƒT > ECOFF). Proportions of target attainment for the MIC-based targets were compared with attainment using clinical breakpoints or PK/PD breakpoints. Treatment response was defined as clinical resolution at day 7 (No persisting signs or symptoms of infection).

**Results:**

We included 98 patients with a median age of 72 years. The most common microbiological finding was *Escherichia coli* (63%) followed by *Klebsiella pneumoniae* (12%). Of all patients, 77/98 patients (79%) attained 100% ƒT > 4MIC and 80/98 (82%) attained 100% ƒT > ECOFF, compared with 57/98 (58%) using 100% ƒT > EUCAST clinical breakpoints. Clinical resolution at day 7 was significantly associated with target attainment applying the target 100% ƒT > 4MIC (*p* = 0.013), but this was not the case when 100% ƒT > ECOFF was applied (*p* = 0.50).

**Conclusions:**

In our material, higher target attainment rates were seen using targets derived from actual MICs, compared to EUCAST clinical breakpoints. Attaining 100% ƒT > 4MIC was associated with resolution of infection, but the latter finding should be interpreted cautiously.

## Introduction

Bloodstream infections (BSIs) are a major reason for sepsis and hospitalization and are associated with significant morbidity and mortality [1, 2, 3, 4]. Gram-negative bacteria may be associated with a higher mortality than Gram-positive bacteria and can be difficult to treat [5]. The most common group of antibiotics used for treatment of BSIs are beta-lactams with broad-spectrum activity against Gram-negative bacteria [6, 7].

The bactericidal effect of beta-lactams depends on the time the unbound antibiotic concentration remains above the minimum inhibitory concentration (MIC) of the bacteria, defined as ƒT > MIC [8]. Suboptimal exposure of the antibiotic at the site of infection may lead to poor outcomes and to the development of antimicrobial resistance [9, 10]. High levels can, for some antimicrobials, lead to toxicity as well as other collateral effects. Therapeutic drug monitoring (TDM) for antibiotics is today most often used to avoid toxicity in drugs with a narrow therapeutic window, such as aminoglycosides, but has increasingly been suggested for beta-lactams as well, in the latter case to ensure target attainment [11]. The optimal beta-lactam PK/PD target to achieve clinical cure in critically ill patients remains undefined [12]. An exposure target of beta-lactam unbound plasma concentration above the MIC for the entire dosing interval (100% ƒT > MIC) has been advocated to ensure that 40–70% ƒT > 4 MIC is achieved in severe infections [13]. In a recent position paper, it is stated that most studies in critically ill patients use the target of 100% ƒT > MIC [14], where MIC is generally defined by PK/PD breakpoints or clinical breakpoints for bacteria intended for treatment coverage. When the actual or tentative species is identified but the there is no MIC for the antibiotic used, species-based clinical breakpoints or antibiotic-based PK/PD targets are generally applied, which is the case in most clinical studies [15, 16].

When the infecting bacterium is known, it is possible to associate a MIC-related antibiotic exposure with the outcome of therapy. In such a case, the choice of antibiotic dose, dosing interval and mode of administration will be influenced by the chosen target [16, 17]. An actual MIC-value, however, can be deceptive to use, due to bacterial population heterogeneity as well as methodological imprecision. Accordingly, different targets, derived from an actual MIC-value may be suggested, such as the epidemiological cut off (ECOFF), when applicable, or 4xMIC. Applying an ECOFF target requires that the isolate has an MIC and/or a zone diameter within the suggested wild-type population. The MIC distributions and ECOFFs are determined and published by The European Committee on Antimicrobial Susceptibility Testing(EUCAST), with regular updates [18]. A different, but less supported, view is to include the methodological imprecision when an actual MIC is applied and set the target at four times the actual MIC.

In this study, the primary objective was to compare target attainments for two MIC-derived targets, based on actual MICs; 100% ƒT > 4MIC and 100% ƒT > ECOFF, with target attainments based on species related clinical breakpoints, in a cohort of patients with Gram-negative bacteremia. As a secondary objective, the association between clinical outcome and target attainment for the respective target, was investigated.

## Methods

### Study design and setting

This was a prospective, single-center, observational study performed at Skåne University Hospital, Malmö. Patients were included from May 2020 to January 2024. Clinical trial number: not applicable.

### Participants

All positive blood cultures are notified daily to the infectious disease consultant at Skåne University Hospital, Malmö. Adult patients (> 18 years) with a Gram-negative blood stream infection that had received at least four doses of either cefotaxime, piperacillin-tazobactam or meropenem were eligible for participation. If a pathogen was resistant to the antibiotic prescribed, the patient was not included in the study. In cases of polymicrobial bacteremia, all bacteria had to be susceptible to the antibiotic for inclusion. The inclusion used convenience sampling depending on research staff availability.

### Antibiotic treatment

Antibiotic treatment initiation, choice of agent, dose and potential adjustments was determined by the treating physician with possible input from an infectious disease consultant and was performed fully independent of the study. TDM of beta-lactam antibiotics was not part of clinical routine during the study and the concentration estimates were not available to the treating physicians (and were thus not part of dose adjustments).

### Blood sampling

Five ml of blood was sampled in serum tubes by qualified staff directly prior to the next antibiotic dose (trough value), after at least four doses (to approximate steady state) had been administered [14]. The blood sample was immediately put on ice before transferred to the clinical biochemistry department where the blood was left on ice to congeal for 30 min and then centrifuged at 4 °C for 10 min at 2000 g, aliquoted and stored frozen in -80 °C until further analysis. Samples were analyzed in batches.

### Antibiotic concentration analysis

Cefotaxime, piperacillin and meropenem were analyzed by Liquid Chromatography tandem Mass Spectrometry (LC-MS/MS) in positive mode. The method was validated using FDAs criteria, at the department of clinical chemistry, Skåne University Hospital, Lund.

The method uses protein precipitated with methanol for sample clean-up. All analytes were quantitated using deuterated isotopes as internal standards. Limit of quantitation (LOQ) was set to 1.0 mg/L, with a precision of 7–11%, and with an upper limit at 256 mg/L.

Samples with concentrations below LOQ were reanalyzed after a dilution of calibrators 10 times, which gave an extended LOQ of 0.10 mg/L.

To estimate the free concentration of the antibiotics, the following equations were used:

Cefotaxime: Free unbound concentration = 0.6 x Total concentration [19].

Piperacillin: Free unbound concentration = 0.7 x Total concentration [20].

Meropenem: Free unbound concentration = 0.98 x Total concentration [21].

### Blood cultures

Blood cultures were taken to a blood culture cabinet and analysed according to clinical routine, independent of the study. The blood cultures usually consisted of an aerobic and anaerobic bottle, that were analyzed with the BACTEC FX system (Becton-Dickinson). In case of a positive signal gram stain and microscopy was performed and the clinician in charge of the patient was informed. Species were identified using MALDI-TOF MS (Bruker Daltronics, Bremen, Germany) on material from the culture bottles, and the material was inoculated on agar plates that were incubated overnight. Antimicrobial susceptibility testing was performed using the EUCAST disk-diffusion method, and susceptibility was interpreted according to EUCAST breakpoints.

### MIC

As a part of the study, an MIC for the antibiotic used in clinical treatment was determined for all bloodstream isolates in the study cohort. Testing of aerobic bacteria was performed on Müller-Hinton agar (Oxoid), supplemented with defibrinated horse blood and beta-NAD for fastidious species, and inoculated with a bacterial suspension of 0.5 McFarland. Anaerobic susceptibility testing was performed on Fastidious Anaerobe Agar supplemented with defibrinated horse blood and inoculated with a bacterial suspension of 1 McFarland. E-test strips (BioMériuex) were used according to the recommendations of the producer.

### ECOFFs

For all isolates, all MICs and available zone diameters were compared to EUCAST wild-type distributions, and for isolates with MICs and zone diameters within the wild-type distribution, the EUCAST ECOFF was applied. For species without published ECOFFs for the antibiotic used, and for isolates defined as non-wild-type by the distributions, four times the MIC was applied as the target.

### EUCAST clinical breakpoints

EUCAST clinical breakpoints were applied according to published recommendations for the species identified. This was 1 mg/L for cefotaxime, 8 mg/L for piperacillin/tazobactam and 2 mg/L for meropenem, with the exception of *Neisseria gonorrhoeae* treated with cefotaxime, where 0.125 mg/l was used. If a EUCAST clinical breakpoints was not defined, priorly suggested EUCAST non-species related breakpoints where used [15].

### Definitions and outcome measures

Actual MIC values were obtained from all bacteria growing from blood cultures and compared with free antibiotic concentration. The primary endpoints were defined as the proportion of patients with target attainment of MIC-derived targets (100% ƒT > 4MIC and 100% ƒT > ECOFF, respectively) compared with target attainment applying EUCAST clinical breakpoints. As a secondary outcome, we assessed the association between individual target attainment for the two MIC-derived targets and clinical resolution at day 7, defined as no persisting signs or symptoms of infection after day 7 after initiation of antibiotic treatment [22, 23]. In cases with more than one Gram-negative bacterium, we based the analysis on the bacterium with the highest MIC to the antibiotic studied.

### Baseline characteristics

Study data was collected prospectively during the inclusion period and managed using REDCap electronic data capture tools hosted at Lund university [24, 25].This included demographic data on sex, age, BMI, comorbidities (Charlson comorbidity index) and site of infection. Estimated glomerular filtration rate (eGFR), was calculated using the Cockcroft–Gault equation. Disease severity was assessed trough the National Early Warning Score (NEWS2) with the highest value on day of inclusion. The only laboratory data collected was CRP (C-Reactive Protein) on day of inclusion.

### Statistical analysis

Baseline characteristics data are presented as medians with interquartile ranges for continuous variables and percentages for categorical variables. The association between clinical resolution at day 7 and individual target attainment using MIC-derived targets was tested using a Chi-squared test. A secondary analysis of this assessment was performed excluding cases with more than one antibiotic with clinical effect against the case bacterium. Another secondary analysis was performed with only *Enterobacteraciae* involded. A p-value of < 0.05 was considered statistically significant. Analyses and figures were done in SPSS (IBM SPSS Statistics Version 27) or in R Statistical Software (v4.2.2, R Core Team (2023). A Language and Environment for Statistical Computing. R Foundation for Statistical Computing, Vienna, Austria. https://www.R-project.org).

### Compliance with ethical standards

This study was performed in line with the principles of the Declaration of Helsinki and was approved by the Swedish Ethical Review Authority (2019–03067). Written informed consent was obtained from the patient. If the patient was incapacitated due to their acute illness, the patient´s next of kin was informed about the study and could opt-out of participation. In included patients who regained mental capacity, written informed consent was sought after discharge. The authors declare no conflicts of interest.

## Results

We included 98 patients with Gram-negative bacteremia (51 treated with cefotaxime, 28 treated with piperacillin/tazobactam and 19 treated with meropenem) between May 2020 and January 2024. The median age of patients was 72 years, 50% were females and median BMI was 27. Median Charlson Comorbidity index was 5and median NEWS2 at day of inclusion was 5. The most common site of infection was urinary tract (52%) followed by abdominal (28%). Patients’ demographics are presented in Table 1. *E. coli* was the most common Gram-negative bacteria (63%) followed by *Klebsiella pneumoniae* (12%), Table 2. Eleven patients had more than one bacterium in the bloodstream at the time of inclusion.

**Table 1:** Patient demographics

	Patients (*n* = 98)
Male sex	49 (50%)
Age, year	72 (63–80)
BMI	27 (23–30)
NEWS2	5 (2–6)
Site of infection	
- urinary	51 (52%)
- abdominal	27 (28%)
- unknown	10 (10%)
- lung	4 (4%)
- other	3 (4%)
- skin/soft tissue	2 (3%)
- intravascular catheter	1 (1%)
Creatinine µmol/L	87 (66–105)
eGFR mL/min	75 (50–102)
CCI	5 (3–6)
CRP	133 (76–229)

Data are presented as n (%) or median (interquartile range)

Abbreviations: BMI, body mass index. NEWS2, national early warning score. eGFR, estimated glomerular filtration rate. CCI, charlson comorbidity index. CRP, c- reactive protein. NEWS2, the highest value on day of inclusion

**Table 2:** Microbiological findings

Escherichia coli	62 (63%)
Klebsiella pneumoniae	12 (12%)
Klebsiella oxytoca	7 (7%)
Bacteroides fragilis	3 (3%)
Enterobacter cloacea	3 (3%)
Pseudomonas aeruginosa	3 (3%)
Proteus mirabilis	2 (2%)
Eikenella corrodens	1 (1%)
Hemophilus influenzae	1 (1%)
Moraxella nonliquefaciens	1 (1%)
Neiseeria gonorrhoeae	1 (1%)
Klebsiella aerogenes	1 (1%)
Bacteroides hominis	1 (1%)

Data are presented as *n* (%)

Most patients were on ‘standard dose’ with intermittent injections for the studied antibiotic, (standard dose = 4 g q8h for piperacillin/tazobactam, 1 g q8h for meropenem and cefotaxime). 16 patients were on adjusted dose, for cefotaxime 2 patients were on 1 g q12h, 5 patients on 2 g q8h and 1 patient on 1000 g q6h. For piperacillin 3 patients where on 4 g q6h and for meropenem one patient on 0,5 g q12h, one on 0,5 g q8h, two on 1 g q6h and one 2 g q8h. The median concentrations, in mg/L were 1.3 (0.5–4.1) for cefotaxime, 8.0 (2.7–13.8) for piperacillin and 4.8 (2.0-8.4) for meropenem. Nine patients were treated with a combination of antibiotics (2 Trimethoprim-sulfamethoxazole, 4 metronidazole, one each of aminoglycoside, fluoroquinolones and ampicillin). Five of these patients had a second antibiotic effective against the culprit bacterium (2 Trimethoprim-sulfamethoxazole, one each of aminoglycoside, fluoroquinolones, ampicillin).

Zone diameters for the antibiotic studied were available in 73/98 (75%) cases. Only one case was non-wild type, an *E. coli* with an MIC of 0,5 mg/L treated with cefotaxime were 4xMIC was applied. Three bacteria (*Eikenella corrodens*,* Moraxella nonliquefaciens*,* Bacteroides hominis*) did not have a defined wild-type distribution, and in these cases 4xMIC was applied. Extended Spectrum Beta-Lactamase (ESBL)-production was identified in six bacterial isolates in the study.

### Target attainment using MIC-derived targets

**Fig. 1:**
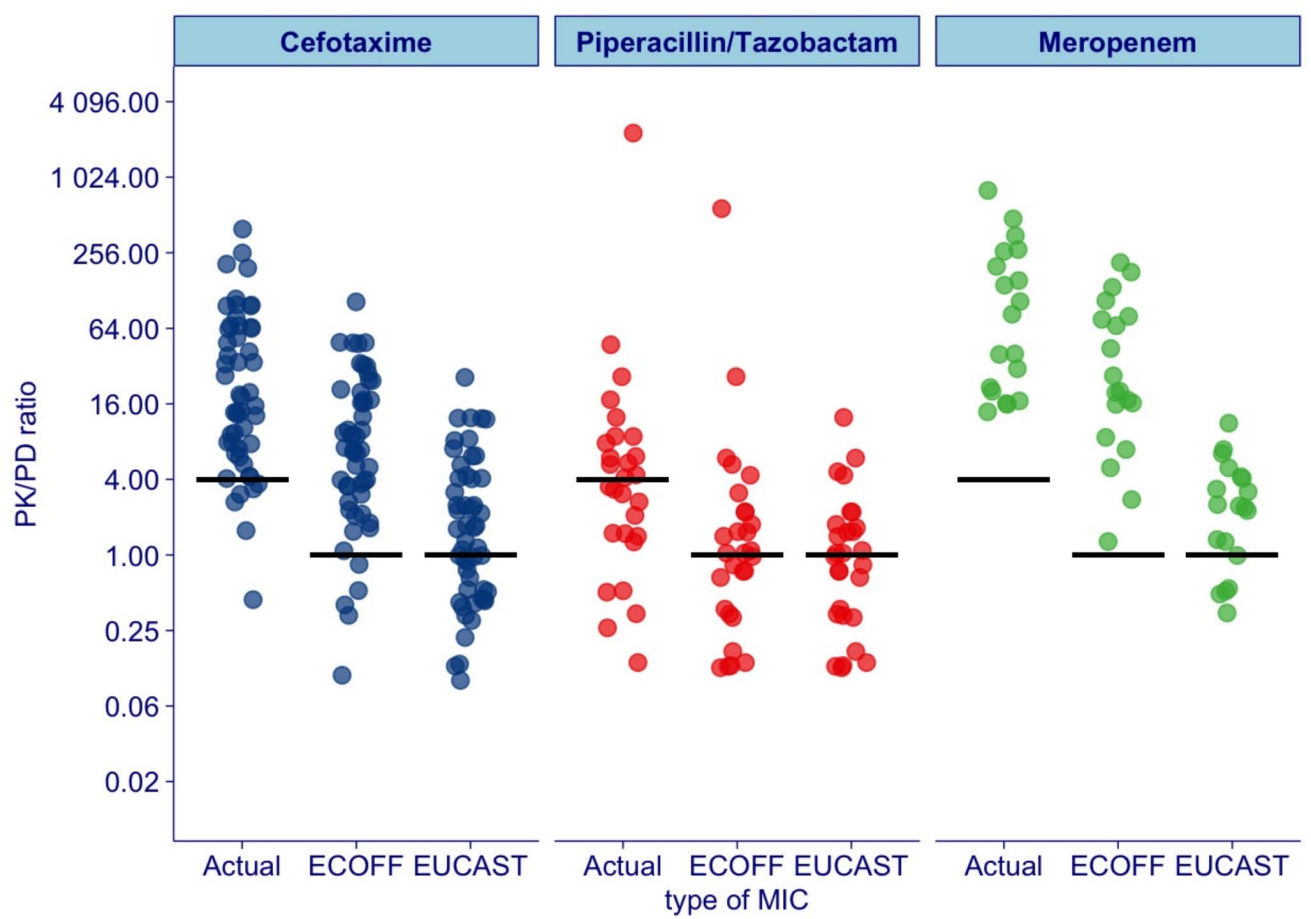
PK/PD ratio by antibiotic and MIC Values (Four times MIC actual, ECOFFs and EUCAST clinical breakpoints). PK/PD ratio = the ratio of free serum concentration / Four times MIC actual, ECOFFs and EUCAST clinical breakpoints of each antibiotic, in trough samples. The lines represent target attainment. Target attainment for MIC Actual was set to100% ƒT > 4MIC, PK/PD ratio > 4. Target attainment for ECOFFs and MIC EUCAST clinical breakpoints was set to 100% ƒT > MIC, PK/PD ratio > 1, respectively

Of all patients, 77 (79%) attained 100% ƒT > 4MIC (Table 3; Fig. 1). Separated for individual antibiotics, 100% ƒT > 4MIC was attained in 44 (86%) treated with cefotaxime, 14 (50%) treated with piperacillin and 19 (100%) treated with meropenem.

**Table 3:** Antibiotic data for pharmacokinetic/pharmacodynamic targets MIC actual

Dosing and PK/PD targets	Cefotaxime (*n* = 51)	Piperacillin (*n* = 28)	Meropenem (*n* = 19)	All (*n* = 98)
100% *f* T > MIC, no. (%)	50 (98)	23 (82)	19 (100)	92 (94)
100% *f* T > 4MIC, no. (%)	44 (86)	14 (50)	19 (100)	77 (79)

Data are presented as number (%)

Abbreviations: MIC, minimum inhibitory concentration; PK/PD, pharmacokinetic/pharmacodynamic

Of all patients, 80 (82%) attained 100% ƒT > ECOFF (Table 4; Fig. 1). Separated for individual antibiotics, 100% ƒT > ECOFF was attained in 46 (90%) treated with cefotaxime, 15 (54%) treated with piperacillin and 19 (100%) treated with meropenem.

**Table 4:** Antibiotic data for pharmacokinetic/pharmacodynamic targets ECOFF

Dosing and PK/PD targets	Cefotaxime (*n* = 51)	Piperacillin (*n* = 28)	Meropenem (*n* = 19)	All (*n* = 98)
100% *f* T > MIC, no. (%)	46 (90)	15 (54)	19 (100)	80 (82)
100% *f* T > 4MIC, no. (%)	31 (61)	5 (18)	17 (89)	53 (54)

Data are presented as number (%)

Abbreviations: MIC, minimum inhibitory concentration; PK/PD, pharmacokinetic/pharmacodynamic

**Fig. 2:**
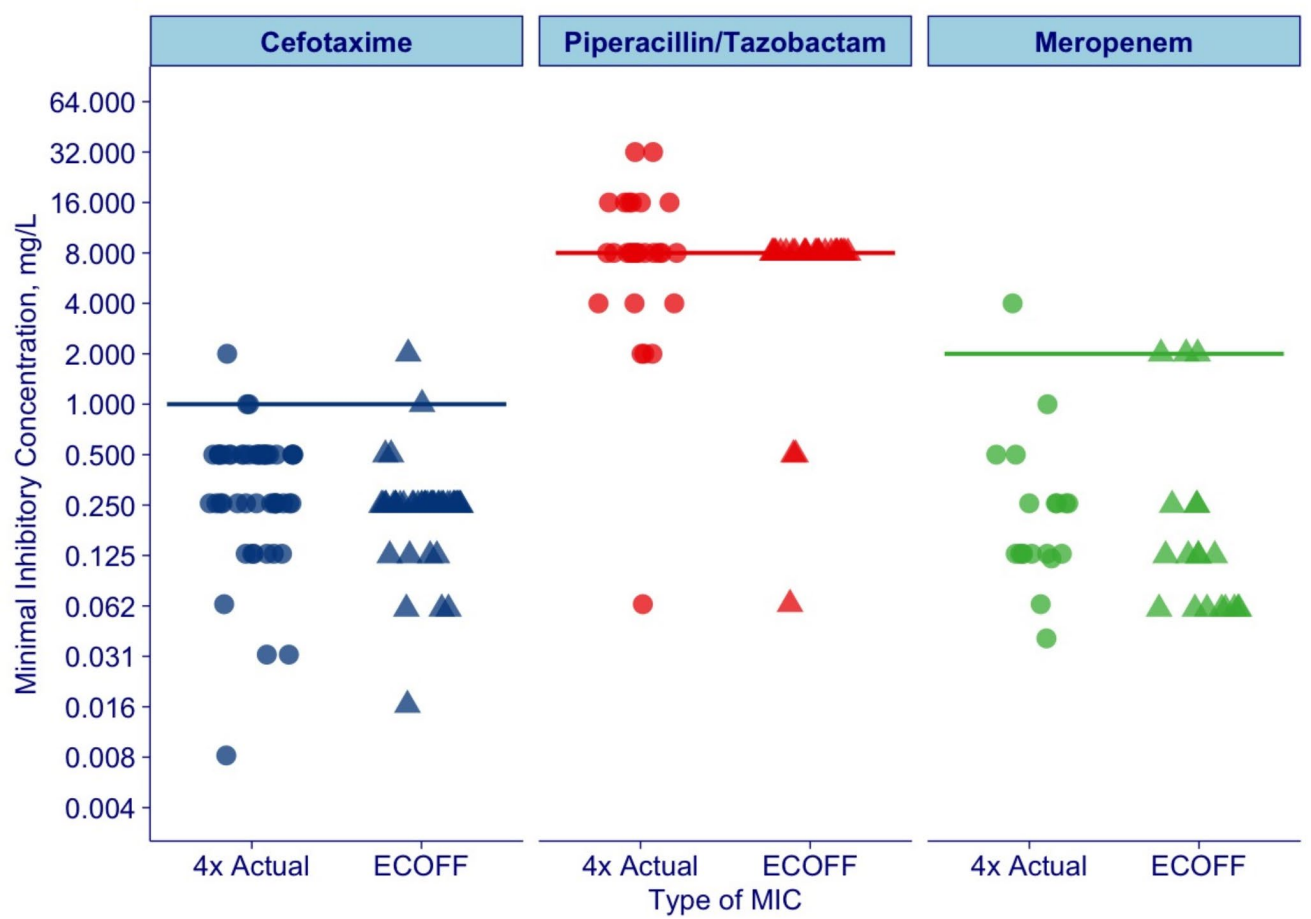
Comparison of MIC targets per antibiotic in mg/L. Three targets were used in the study and presented here, four times MIC actual, ECOFFs and EUCAST clinical breakpoints. The dots represent four times actual MIC values. The triangles represent ECOFFs values applied. The three lines represents EUCAST clinical breakpoints applied (1 mg/L for cefotaxime, 8 mg/L for piperacillin, 2 mg/L for meropenem), except for Neisseria gonorrhoeae treated with cefotaxime, where 0.125 mg/l was used

Targets used for four times MIC and ECOFFs are shown in Fig. 2.

### Target attainment using EUCAST clinical breakpoints

Of all patients, 57 (58%) attained 100% ƒT > Clinical breakpoints (Table 5; Fig. 1). For each antibiotic, target was attained in 29 (57%) treated with cefotaxime, 14 (50%) treated with piperacillin and 14 (74%) treated with meropenem.

**Table 5:** Antibiotic data for pharmacokinetic/pharmacodynamic targets clinical breakpoints

Dosing and PK/PD targets	Cefotaxime (*n* = 51)	Piperacillin (*n* = 28)	Meropenem (*n* = 19)	All (*n* = 98)
100% *f* T > MIC, no. (%)	29 (57)	14 (50)	14 (74)	57 (58)
100% *f* T > 4MIC, no. (%)	15 (29)	4 (14)	6 (32)	25 (26)

Data are presented as number (%)

Abbreviations: MIC, minimum inhibitory concentration; PK/PD, pharmacokinetic/pharmacodynamic

Targets used for EUCAST clinical breakpoints are shown in Fig. 2.

### Clinical outcomes

The 30-day mortality was 3% (3/98 patients). In individuals attaining the target 100% ƒT > 4MIC, clinical resolution was observed in 49/77 patients (64%) compared with 7/21 patients (33%) not meeting this target, (*p* = 0.013). In a secondary analysis, where patients receiving concurrent antibiotic treatment effective against the culprit bacteria were removed (*n* = 5), the association between attaining 100% ƒT > 4MIC and clinical resolution remained significant (*p* = 0.006).

In contrast, attaining 100% ƒT > ECOFF was not associated with a significantly higher rate of clinical resolution (*p* = 0.50). In individuals meeting the 100% ƒT > ECOFF target, clinical resolution was observed in 47/80 patients (59%) compared with 9/18 patients (50%) in the group not reaching this target. This association remained non-significant when patients with concurrent effective antibiotic treatment were removed.

Attaining 100% ƒT > Clinical breakpoints was not associated with significantly higher rate of resolution (*p* = 0.9). In individuals meeting the 100% ƒT > Clinical breakpoints target, clinical resolution was observed in 33/57 patients (58%) compared with 23/41 patients (56%) in the group not reaching this target.

The majority of bacteria in the study were of the family *Enterobacteraciae*, and a secondary analysis was performed with only *Enterobacteraciae* (*n* = 88). In this secondary analysis with only *Enterobacteraciae*, attaining clinical resolution and 100% ƒT > 4MIC still remained significant (*p* = 0.007) and remained non-significant in the ECOFF and EUCAST group.

## Discussion

In this single-center prospective study we found that in patients with Gram-negative bacteremia, approximately 20% of patients did not meet conservative PK/PD (MIC-derived) targets, in presumed steady state, while this number was 42% when EUCAST clinical breakpoints, that can be referred to as standard targets, were applied.

This is not the first study to show that patients with severe infections are potentially underdosed if current PK/PD targets are applied [6, 26, 27]. In the present study, we had access to actual MICs for the treatment antibiotic in all cases, and full comparisons between MIC-derived targets and clinical breakpoints targets could be made. The results suggest that fewer patients with Gram-negative bacteremia in our setting would be categorized as underdosed when MIC-derived targets are applied. Applying such targets could lower the risk of treatment toxicity and unwanted side effects [12]. The main challenge with underdosing in our material, even when actual MIC-derived targets were determined, was observed in patients treated with piperacillin-tazobactam. When comparing ECOFFs with clinical breakpoints they are generally lower for cefotaxime and meropenem resulting in higher target attainment when using ECOFF-based targets. In contrast, ECOFFs and clinical breakpoints are generally equal for piperacillin, with no margin between the upper limit of the wild-type population and PK/PD-based concentration distributions. Previous studies have also shown that underdosing is a concern in critically ill patients treated with piperacillin-tazobactam specifically [28, 29, 30]. In most ICU studies on beta-lactam antibiotic concentrations, higher targets are used since the causative agents is often unknown and the target is set to worst case scenarios [31, 32].

There is still no consensus in the medical or scientific community as what target is the most appropriate for beta-lactams in severe infections, as infection resolution will depend on different factors, such as treatment delay, infection severity, patient frailty and trained immunity, to name just a few. A target of unbound concentrations of four times above the MIC throughout the entire dosing interval (100% ƒT > 4MIC) has been suggested to ensure maximal bacterial killing, prevent bacterial regrowth and facilitate positive clinical outcomes as seen in our study [33]. To estimate target attainment for potentially difficult to treat bacteria, targets are often derived from MICs of a representative difficult to treat bacteria such as *Pseudomonas aeruginosa*, for Gram-negative infections and/or EUCAST nonspecific clinical breakpoints. As shown in the present study, using clinical breakpoints often lead to reduced target attainment as these breakpoints are higher compared to actual MIC breakpoints. If striving for these targets, doses will need to be increased with, as, of today, no proven benefit, and an increased risk of toxicity. The discriminatory power will then also be reduced as targets don’t accurately reflect the MICs of the causative pathogens and target attainment will be underestimated. EUCAST nonspecific clinical breakpoints, that have been used in many previous studies, have now been removed from the EUCAST webpage. A recent Swedish multicenter study stated that when using worst case scenarios MIC values, when the pathogen is unknown, can be a reason for not attaining target. The authors suggest that ECOFF values based on the MIC of the causing or potentially causing bacterium should be used [34]. Our study also suggests that the use of PK/PD targets derived from clinical breakpoints or worst case scenario pathogens, may lead to unnecessarily high dosages. MIC applied as four times MIC or potentially ECOFFs could preferably be used as target, when the MIC is available, in future studies. In a recent paper on beta-lactam antibiotics and bloodstream infections attaining 100% ƒT > 4MIC was associated with shorter time to negative blood cultures and more negative blood cultures on day 7 [35].

There are several limitations to this study. First, we attained the MIC with E-test and not with broth dilution which is gold standard [36]. E-test it the preferred method at our microbiological lab and is used in clinical practice. We did not have zone diameter in 25 patients due to the method VITEK being used and that gives only S (sensitive) or R (resistant) for those blood cultures. It is reasonable to assume that the 25 bacteria that did not have zone diameter would be wild type. Our study was a single-center study in a low-resistance setting with few difficult-to-treat bacteria, limiting the external validity of the results. Concerning ESBL bacteria in this study all were treated with meropenem and were considered as wild type after discussion with EUCAST representatives. It is challenging to analyze clinical outcomes with a limited study population, due to low statistical power. Our study was not powered to properly assess clinical differences and the result regarding clinical resolution needs to be interpreted with caution. Furthermore, current dosage regimens for cefotaxime for life-threatening infections is 2 g q8h and for piperacillin-tazobactam 4q 6 h. Most of our patients were on lower dose regimens, and with the higher dose regime today likely more patients would attain targets. Finally, our study was performed in a setting with convenience sampling, which is not necessarily representative of a full population of Gram-negative bacteremia.

Most previous studies on antibiotic concentrations and target attainment have studied mortality as an outcome. No study has yet convincingly demonstrated that TDM for beta-lactam reduces mortality [37]. In two recent meta-analyses, significant advantages of TDM regarding clinical cure, microbiological cure and lower risk of treatment failure were found [38, 39]. Underdosing of antibiotics is more common in younger male patients with augmented renal clearance, a group with lower general risk for mortality due to bacterial infections. It is possible that resolution of infection is therefore a reasonable outcome measure in further studies of beta-lactam TDM.

## Conclusion

This study demonstrates two important findings; that target attainment is not 100% in patients with Gram-negative bacteremia and that in a low-resistance setting, beta-lactam PK/PD targets derived from actual MICs provide improved rates of target attainment compared with generic targets based on EUCAST clinical breakpoints.

## Data Availability

No datasets were generated or analysed during the current study.
